# Clinical, Translational and Experimental Pharmacotherapeutics Advances

**DOI:** 10.3390/jcm12031136

**Published:** 2023-02-01

**Authors:** Zhiyao He, Lei Yu, Zhijie Xu, Chenyu Sun

**Affiliations:** 1 Department of Pharmacy, West China Hospital, Sichuan University, Chengdu 610041, China; 2 Key Laboratory of Drug-Targeting and Drug Delivery System of the Education Ministry and Sichuan Province, Sichuan Research Center for Drug Precision Industrial Technology, West China School of Pharmacy, Sichuan University, Chengdu 610041, China; 3 Department of Pathology, Xiangya Hospital, Central South University, Changsha 410008, China; 4 Department of Thyroid and Breast Surgery, The Second Hospital of Anhui Medical University, Hefei 230601, China; 5 AMITA Health Saint Joseph Hospital Chicago, Chicago, IL 60657, USA

Pharmacotherapeutics is the prevention and treatment of ailments, whether disorders or diseases, with medication. In this Special Issue, we divide pharmacotherapeutics into three parts according to the stage of drug research and use, namely experimental pharmacotherapeutics, translational pharmacotherapeutics and clinical pharmacotherapeutics. The phrase experimental pharmacotherapeutics refers to the experimental and basic research for purpose of pharmacotherapeutics. Translational pharmacotherapeutics is a novel concept, which is devoted to translating novel theranostics from bench to bedside. Clinical pharmacotherapeutics refers to the rational use of drugs to diagnose, prevent and treat diseases in clinical practice. These three parts promote each other and develop together, fueling the progress of pharmacotherapeutics. 

Pharmacotherapeutics is a field that has been absorbing novel technologies and lasting innovation, such as the design and discovery of new targets and new molecular entities, the establishment of new platforms for efficacy and safety evaluation, and new regimens of rational use in clinical practice. At the same time, this distinction also reflects the disciplinary characteristics of pharmacotherapeutics, a field which contains experimental, translational and clinical perspectives. There are many innovations and entry points, and their new advances need to be reported and displayed. 

Clinical pharmacotherapeutics, serviced by experimental and translational pharmacotherapeutics, is the application of pharmacy knowledge together with information about the disease for management of health/ illness. Guiding clinical rational drug use and improving medication therapy management contribute to achieving the optimal level under the five rights of medication administration. Novel clinical pharmacotherapeutic applications also need to be explored and reported. 

Devoting to rapidly reporting the latest advances, we also hope that this Special Issue can provide a comprehensive research frontier in this field and present an in-depth research progress of pharmacotherapeutics to readers.

